# Laboratory and Field Performance Evaluation of High-Workability Ultra-Thin Asphalt Overlays

**DOI:** 10.3390/ma15062123

**Published:** 2022-03-14

**Authors:** Jinquan Wang, Jia Sun, Sang Luo, Qiang Li

**Affiliations:** 1 School of Transportation, Southeast University, Nanjing 211189, China; 230218924@seu.edu.cn (J.W.); 101011363@seu.edu.cn (S.L.); 2 Ningbo Hangzhou Bay Bridge Development Co., Ltd., Ningbo 315033, China; 3 School of Civil Engineering, Nanjing Forestry University, Nanjing 210037, China; liqiang2526@njfu.edu.cn

**Keywords:** ultra-thin asphalt overlay, high workability, high- and low-temperature performance, moisture damage resistance, field road performance

## Abstract

The defects of poor workability and inadequate pavement performance of the ultra-thin asphalt overlay limited its application in the preventive maintenance of pavements. In this study, a high-workability ultra-thin (HWU) asphalt overlay scheme was proposed. A high-strength-modified asphalt binder and an optimized HWU-10 gradation were used to prepare the HWU asphalt mixture and explore its laboratory performance. Furthermore, the HWU asphalt mixture was used for the test road paving. Based on the field performance test results before and after the test road for one year of traffic operation, the application performance of the HWU asphalt mixture and styrene-butadiene-styrene (SBS)-modified asphalt mixture was compared and analyzed. The results showed that the HWU asphalt mixture possessed satisfactory laboratory pavement performance, and its high-temperature stability and moisture damage resistance were better than those of the SBS-modified asphalt mixture. The asphalt mixture prepared using HWU-10 gradation was easily compacted and showed good workability. After one year of operation, all field performance of the ultra-thin overlay paved with HWU asphalt mixture met the specification requirements, but its flatness and skid resistance decreased. It is worth mentioning that the HWU asphalt mixture was significantly better than the SBS-modified asphalt mixture in terms of performance degradation resistance and rutting resistance. The studies to enhance the road intersection pavement performance and ensure the homogeneous dispersion of polyester fibers in the asphalt mixture will be considered in the future.

## 1. Introduction

The rapid economic growth has promoted the vigorous development of the transportation industry, and the sudden increase in traffic volume and vehicle load has undoubtedly caused the aggravation of road surface defects [1]. In asphalt pavement structures, the surface layer is directly in contact with tires and the atmosphere, such that it is more susceptible to damage than other pavement structural layers under the complicated influence of vehicle loads, solar radiation, and moisture. In general, asphalt pavement surface layer will show defects such as rutting, cracks, and potholes after 5–10 years of service. The decline in pavement functionality will affect the driving experience and safety, as well as reduce the road integrity and service life [2]. Therefore, it is necessary to carry out preventive maintenance on the surface layer of asphalt pavement to ensure the comfort and safety of driving by providing the pavement with excellent flatness, rutting resistance, and water damage resistance.

The commonly used preventive maintenance techniques for asphalt pavements include slurry seal, micro-surfacing, fog seal, and ultra-thin overlay [3,4,5]. The slurry seal adopts dense grading, cold-mixing, and cold-paving process, so it has excellent waterproof performance and environmental protection. However, this technique is only applicable to the repair and maintenance of pavements with minor damage, and its long curing time leads to prolonged traffic opening time [6,7]. Micro-surfacing is an improved technology based on the slurry seal, which has better rutting resistance and durability [8,9]. It is noteworthy that the design life of the micro-surfacing is only 3–5 years, and the pavement maintained with this technology has the drawbacks of high traffic noise [10]. The addition of the fog seal requires the original pavement to continue good overall performance, and it can improve the water resistance, flatness, and skid resistance to some extent. Unfortunately, this method only applied a super-thin film coating on the surface layer of the pavement, which has an insignificant effect on enhancing the structural strength and the integrity of the pavement [11]. Consequently, the fog seal technology is only applicable to pavements with minor cracks and early stage defects. Thereafter, a method with good maintenance efficiency as well as structural strength improvement is indeed to be proposed. The ultra-thin asphalt overlay has thus become the most widely used preventive maintenance technology due to its wide applicability, short construction period, and long service life. Simultaneously, it adopts gap gradation and special large-scale paving equipment to ensure skid resistance and construction quality [12,13,14].

The ultra-thin asphalt overlay (Nova Chip) was proposed and developed in France in 1986 and was widely used in Germany and the United States in the 1990s [15,16]. Subsequently, with the popularity and application of ultra-thin overlay technology, scholars have conducted a number of research studies on it. Ding et al. developed a highly durable ultra-thin overlay and analyzed its durability [17]. The results showed that the asphalt mixture had excellent aging performance, fatigue resistance, and durability compared with that of virgin asphalt and styrene-butadiene-styrene (SBS)-modified asphalt mixtures. In the meanwhile, a corresponding durability evaluation system was proposed. Yu et al. proposed a novel scheme called high ductility ultra-thin friction course (HUFC). High-viscosity asphalt binder and HUFC-8 gradation were selected to test the laboratory performance of the HUFC asphalt mixture. The results showed that the HUFC asphalt mixture had excellent mechanical properties and skid resistance, and the paving technology had the potential to be applied on a large scale [18]. Cui et al. developed an ultra-thin wear course (UTWC) asphalt mixture and compared it with a conventional asphalt mixture. The results showed that the UTWC asphalt mixture possessed better high-temperature stability and skid resistance and that its low-temperature crack resistance and moisture damage resistance were satisfactory. Moreover, the researchers also studied the porous ultra-thin asphalt overlay [19]. Liu et al. prepared a high-viscosity-modified asphalt for porous ultra-thin asphalt overlays using different gradations and modifiers, and studied the performance of binders and mixtures. The results showed that the comprehensive performance of high-viscosity-modified asphalt was ideal. Compared with the dense gradation, the open-graded asphalt mixture had better drainage performance and skid resistance [20,21]. Hu et al. designed an OGFC-5 porous asphalt ultra-thin overlay and evaluated its performance. Through the tests, it was found that the moisture damage resistance of OGFC-5 was good, and the increase in fine aggregate content contributed to the improvement of cohesion and reduction in internal friction angle [22]. Accordingly, previous studies have shown that ultra-thin asphalt overlay is a preventive maintenance technology with excellent performance and research value. It is noteworthy that these studies focused on the laboratory performance of ultra-thin overlay asphalt mixtures and did not apply them in engineering and verify their practical application performance through field performance tests. In addition, the mixture used in the ultra-thin asphalt overlay is generally open gradation with a significantly large proportion of coarse aggregates. This leads to the asphalt mixture being difficult to roll compact. The poor construction workability undermines the compactness of the ultra-thin asphalt cover layer, resulting in its pavement performance being limited to some extent.

The objective of this study is to propose a high-workability ultra-thin (HWU) asphalt overlay scheme that ensures both good workability and pavement performance. The gradation of the mixture was modified to obtain an optimized gradation, designated HWU-10. The high-strength-modified asphalt binder and polyester fibers were used to prepare the HWU asphalt mixture and then used to pave the ultra-thin overlays of the test roads. Combined with laboratory and field performance tests to verify its engineering application performance, which had a guiding significance for the promotion and application of HWU asphalt overlay. Firstly, the high-strength and SBS-modified asphalt binders were used to prepare the mixtures, the mix proportion design was carried out, and the optimum asphalt contents were determined. Secondly, through a series of tests, the laboratory properties of the mixtures, such as high-temperature stability, low-temperature crack resistance, moisture damage resistance, and skid resistance, were compared and analyzed. Finally, HWU and SBS-modified asphalt mixtures were used to pave ultra-thin asphalt overlays on the test roads. The flatness, skid resistance, compaction degree, permeability, and rutting depth of the test roads before and after one year of opening traffic were compared based on a series of field tests to evaluate their engineering application performance.

## 2. Background of the Field Test Pavements

**Figure 1:**
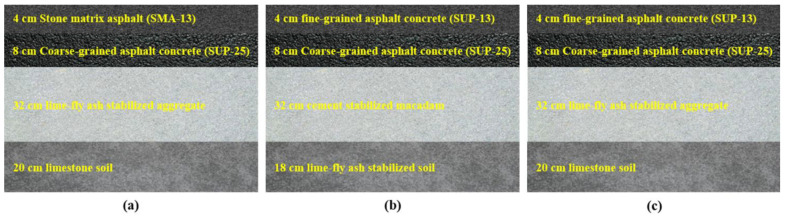
Original structure of the test roads: (**a**) test road 1; (**b**) test road 2; (**c**) test road 3.

The test roads paved in this study were all located in Suzhou city, Jiangsu Province in China. As shown in Figure 1, test road 1 was a first-class highway with the original surface layer of 4 cm stone matrix asphalt (SMA-13) and 8 cm coarse-grained Superpave asphalt concrete (SUP-25), the base layer of 32 cm lime-fly ash stabilized aggregate, and the subgrade of 20 cm limestone soil. Test road 2 was a second-class highway. The original surface layer of 4 cm fine-grained Superpave asphalt concrete (SUP-13) and 8 cm coarse-grained Superpave asphalt concrete (SUP-25), the base layer of 32 cm cement-stabilized macadam, and the subgrade of 18 cm lime-fly ash-stabilized soil. Test road 3 was a second-class highway, the original surface layer of 4 cm fine-grained Superpave asphalt concrete (SUP-13) and 8 cm coarse-grained Superpave asphalt concrete (SUP-25), the base layer of 32 cm lime-fly ash-stabilized aggregate, and the subgrade of 20 cm limestone soil. The ages of original surface layers in test roads 1, 2, and 3 were 13, 14, and 16 years, respectively.

**Figure 2:**
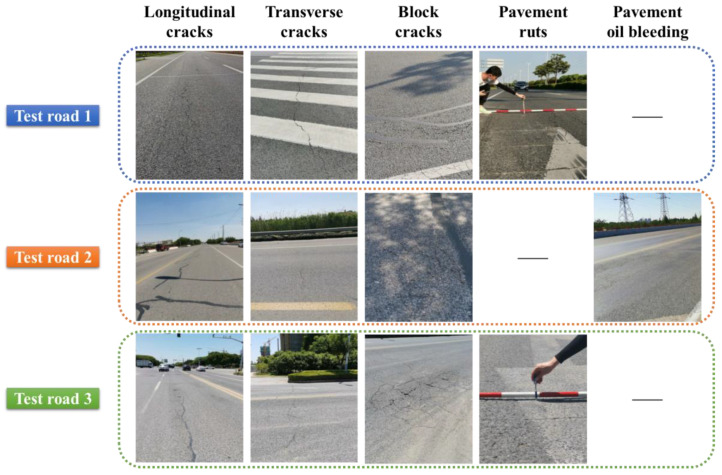
Original defects of the test roads.

**Figure 3:**
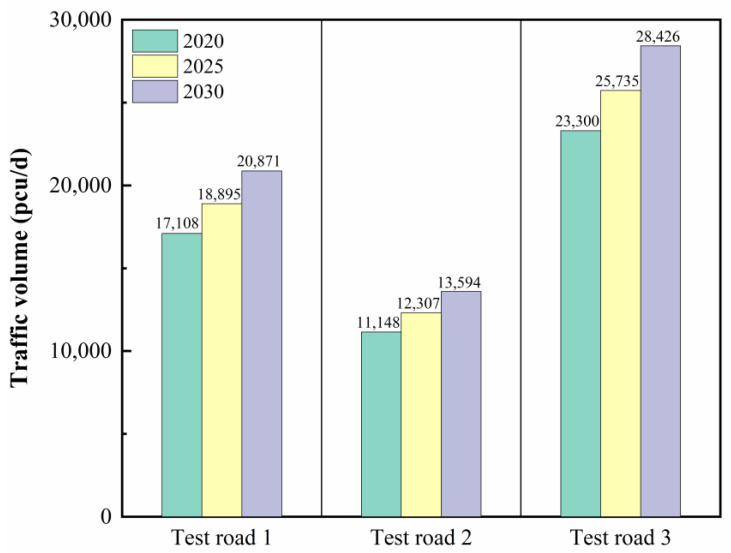
Current status and prediction of traffic volume on the test roads.

The original types and distribution of defects on the test roads were investigated, and the traffic volume of each test road was counted and predicted. As shown in Figure 2, the damage types of test road 1 were mainly transverse, longitudinal, and block cracks, ruts existed in local sections, ruts and cracks were serious at junctions, and the overall damage level of the road was slight. Test road 2 was dominated by transverse, longitudinal, and block cracks, and oil bleeding existed in local sections, and the overall damage was slight. The defects on test road 3 were mainly cracks (longitudinal, transverse, and block cracks) and ruts, with sinkholes and potholes in local sections. The overall damage of test road 3 was more severe than that of test roads 1 and 2. Figure 3 illustrates the current status of traffic volume on each test road and the predicted results for the future. Specifically, test road 3 had the highest traffic volume while test road 2 had the lowest traffic volume of 23,300 and 11,143 pcu/d. Influenced by the economic development and the improvement of the living standard, the predicted traffic volume of each road section is gradually increasing with time. In 2030, the predicted traffic volumes for test roads 1, 2, and 3 will reach 20,871, 13,594, and 28,426 pcu/d, respectively.

## 3. Methodology

### 3.1. Raw Materials

In this study, high-strength asphalt binder (Jiangsu Tiannuo Road Material Technology Co., Ltd., Zhenjiang, China) and SBS-modified asphalt binder (Jiangsu Donghua Construction Material Co., Ltd., Suzhou, China) were used to prepare HWU and SBS-modified asphalt mixtures, respectively. The high-strength asphalt binder consists of 60–80 penetration grade base asphalt binder, rock asphalt binder, SBS, rutting resistance agent (1.3:2.4)-dibenzylidene sorbitol (DBS), and ultra-thin overlay asphalt modifier. The mass ratio of each component was 60–80 penetration grade base asphalt binder:rock asphalt binder:SBS:DBS:ultra-thin overlay asphalt modifier = 0.883:0.053:0.04:0.017:0.007. The SBS-modified asphalt binder was prepared using linear SBS with 40% styrene content, and the addition amount was 4.5 wt% of the binder. The aggregates used for preparing the mixture include coarse basalt aggregate, fine basalt aggregate, and mineral powder obtained by grinding alkaline limestone stone. In addition, polyester fibers (Yancheng Huawei Road Engineering Fiber Material Co., Ltd., Yancheng, China) were added to the HWU asphalt mixture. The addition of polyester fibers in the asphalt mixture can play a reinforcing and bridging effect on the pavement, which improves the high-temperature stability, moisture damage resistance, and fatigue resistance. However, the addition of polyester fibers leads to an increase in mixing time and aggravates asphalt aging. Simultaneously, polyester fibers tend to absorb asphalt, leading to an increase in the optimum asphalt content of the mixture. Therefore, combined with the field mix proportion design and previous construction experience, its content was determined to be 0.15 wt% of the asphalt mixture without increasing the mixing time. Table 1 and Table 2 illustrate the physical properties of high-strength-modified asphalt binder, SBS-modified asphalt binder, and polyester fiber. Specifications are in accordance with the “Technical Specification for Highway Asphalt Pavement Construction” (JTG F40–2004) [23].

### 3.2. Proportion Design of the Mixture

**Figure 4:**
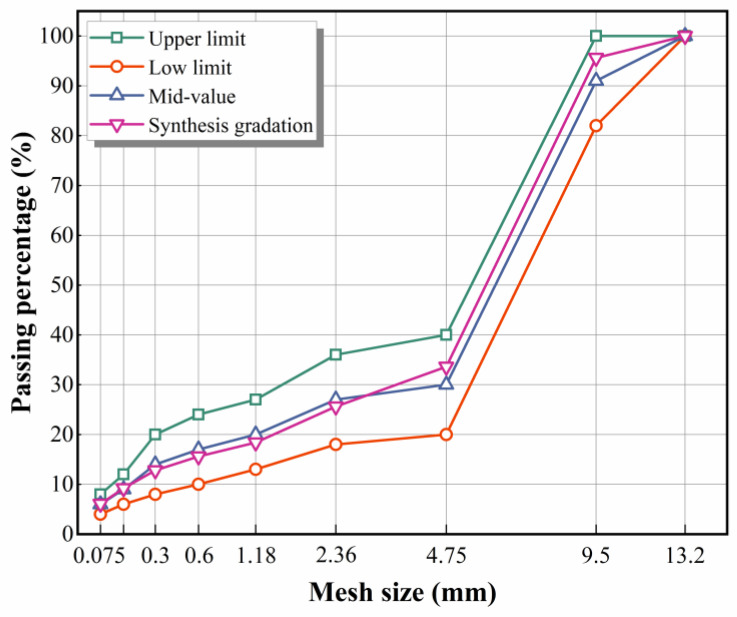
Gradation curves of aggregates used for preparing asphalt mixtures.

The HWU-10 gradation was selected for the asphalt mixture gradation design. The gradation curve of HWU-10 is shown in Figure 4, which was within the upper and lower limits of aggregate gradation requirements. The optimum asphalt content of the asphalt mixture was predicted to be 6.0% and varied at ±0.5% intervals to obtain five different asphalt contents. Five groups of Marshall specimens were prepared with a mixing temperature of 160 °C and a compaction temperature of 150 °C. The Marshall stability test was conducted on the specimens to test their bulk density, Marshall stability, and flow value and calculate the void volume, voids in mineral aggregate, and asphalt saturation. According to the Marshall test results, the optimal asphalt contents of HWU and SBS-modified asphalt mixtures were finally determined to be 6.0% and 5.5%, respectively. It can be seen that the optimal asphalt content of the HWU asphalt mixture was higher than that of the SBS-modified asphalt mixture, which was mainly caused by the absorption of asphalt by polyester fibers in the HWU-modified asphalt mixture.

### 3.3. Paving of the Field Test Sections

**Figure 5:**
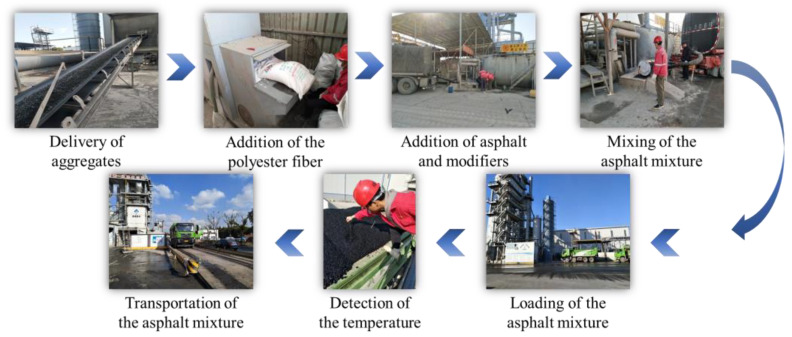
Preparation and transportation procedures of the HWU asphalt mixture.

**Figure 6:**
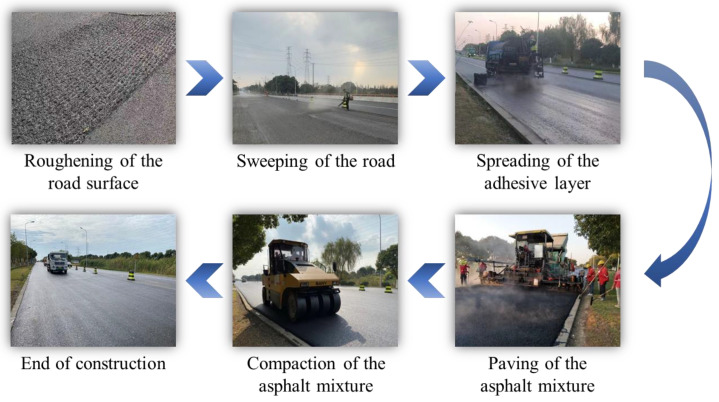
The field construction process of HWU asphalt overlay.

The HWU and SBS-modified asphalt mixtures were used to pave ultra-thin overlays with a thickness of 25 mm on the top layer of the test roads. The SBS-modified asphalt mixture was adopted for test road 1, while the HWU asphalt mixture was applied for test roads 2 and 3. The preparation, transportation, and field construction procedures of the HWU ultra-thin overlay are shown in Figure 5 and Figure 6, whereas the SBS-modified ultra-thin overlay process was similar.

As shown in Figure 5, the processes of preparation and transportation of the HWU asphalt mixture are as follows: (i) The mixing plant addition procedure was confirmed, and the aggregates were transported through automated equipment; (ii) according to the mix ratio, a quantitative amount of polyester fiber was added to the mixing plant; (iii) the high-strength asphalt binder and ultra-thin overlay asphalt modifier were added to the mixing plant; (iv) the HWU asphalt mixture was thoroughly mixed and consolidated; (v) the completed HWU asphalt mixture was loaded using a transport vehicle; (vi) the transport vehicle after loading was inspected, including visual observation of the fibers and temperature detection to ensure that the mixed HWU asphalt mixture had no apparent fiber agglomeration and the temperature met the construction requirements; (vii) the transport vehicle was weighed after completing the quality inspection, and then the HWU asphalt mixture was sent to the construction field.

Figure 6 illustrates the field construction process of the HWU asphalt overlay. Specifically, (i) before paving ultra-thin asphalt overlay, the original pavement was brushed to enhance its interlayer adhesion capacity; (ii) the brushed asphalt pavement was swept clean; (iii) an intelligent asphalt spray truck was used to spray high-viscosity emulsified asphalt, and the sprayed viscous layer oil became a uniform mist, and the spraying quantity was 0.4–0.6 L/m^2^; (iv) after the demulsification of emulsified asphalt and the water evaporation was completed, the paving machine should be used immediately to pave the asphalt overlay to reduce the adverse effects caused by the contamination of the sticky layer. The paving speed was 2.5–3.5 m/min, the paving thickness was 30 mm, and the loose-laying coefficient was 1.2; (v) steel drum rollers were used for initial compaction (rolled 1–2 times), rubber drum rollers were used for re-compaction (rolled 4–5 times), and steel drum rollers were used for final compaction (rolled 2–3 times) to form an ultra-thin asphalt overlay with a thickness of 25 mm; and (vi) after compaction was completed and the construction machinery was retired, traffic could be opened with no overloaded vehicles.

## 4. Test Method

As demonstrated in Table 3, according to the “Standard Test Methods of Bitumen and Bituminous Mixtures for Highway Engineering.” (JTG E20–2011) and “Field Test Methods of Highway Subgrade and Pavement” (JTG 3450–2019) of China [24,25], the rutting test, bending beam test, freeze–thaw splitting tensile strength test, and pendulum test were used to experimentally investigate the high-temperature stability, low-temperature crack resistance, moisture damage resistance, and skid resistance of asphalt mixtures, respectively [26,27,28]. Moreover, the flatness, skid resistance, compaction degree, anti-permeability performance, and rutting depth of the test roads before and after operation were compared by the flatness test, pendulum test, compaction test, permeability test, and rutting depth test. Three parallel trials were conducted for each test.

### 4.1. High-Temperature Stability Test

The rutting test was carried out using an SYD-0719C rutting tester (Shanghai Changji Geological Instruments Co., Ltd., Shanghai, China) with a slab specimen size of 300 mm × 300 mm × 50 mm. Subsequently, the specimen was placed on the same track and repeatedly crushed with a solid rubber wheel to form a groove. The test temperature and the wheel pressure were 60 °C and 0.7 MPa, respectively. The dynamic stability (*DS*) was used as an evaluation index for the rutting resistance of the HWU and SBS asphalt mixtures. The *DS* was calculated according to Equation (1).

(1) $$DS=\frac{\left(t_{2}-t_{1}\right)\times N}{d_{2}-d_{1}}\times C_{1}\times C_{2}$$

where *DS* is the dynamic stability of asphalt mixture, times/mm; *d*_1_ is the deformation of the corresponding time *t*_1_, mm; *d*_2_ is the deformation of the corresponding time *t*_2_, mm; *C*_1_ is the correction factor of the type of testing machine; *C*_2_ is the coefficient of the specimen; and *N* is the round-trip speed of the test wheel, typically 42 times/min.

### 4.2. Low-Temperature Crack Resistance Test

The rutting slab was cut into small beams of 250 mm × 30 mm × 35 mm and tested using a UTM-25 servo-hydraulic multifunctional material testing machine (IPC Global, Boronia, VIC, Australia). The test temperature and the loading rate were −10 °C and 50 mm/min, respectively. The low-temperature crack resistance of asphalt mixtures was evaluated using the maximum flexural strain (*ε*_B_). The *ε*_B_ was calculated according to Equation (2).

(2) $$\varepsilon_{B}=\frac{6\times h\times d}{L^{2}}$$

where *ε*_B_ is the maximum flexural strain of the specimen at the time of damage, μ*ε*; *h* is the height of the interrupted specimen, mm; *L* is the span diameter of the specimen, mm; and *d* is the span deflection of the specimen at the time of damage, mm.

### 4.3. Moisture Damage Resistance Test 

The moisture damage resistance was evaluated based on the freeze–thaw splitting tensile strength test. Standard Marshall specimens with dimensions of 101.6 mm × 63.5 mm were prepared by using the SYD-0702 Marshall compaction instrument (Shanghai Changji Geological Instrument Co., Ltd., Shanghai, China). The specimens were divided into two groups, and one group of specimens was placed in a water bath (Shanghai Qianjun Scientific Instruments Co., Ltd., Shanghai, China) at −18 °C for 16 h, and the specimens were taken out and immediately placed at a constant temperature water bath at 60 °C for 24 h. Then, the two groups of specimens were all immersed in a constant temperature water bath at 25 °C for 2 h. Finally, the specimens were removed and subjected to splitting tests using a Marshall tester (Beijing Hafuda Technology Co., Ltd., Beijing, China) to calculate the freeze–thaw splitting tensile strength ratio (TSR), as shown in Equation (3).

(3) $$TSR=\frac{RT_{2}}{RT_{1}}\times 100$$

where *TSR* is the freeze–thaw splitting strength ratio, %; *RT*_2_ is the splitting tensile strength of the second group of specimens after freezing and thawing, MPa; and *RT*_1_ is the splitting tensile strength of the first group of specimens without freezing and thawing, MPa.

### 4.4. Pendulum Test 

A pendulum coefficient of friction tester (Jiangsu Shuyang Kexing Highway Instrument Co., Ltd., Suqian, China) and a rubber sheet were used to perform slip resistance testing to obtain British pendulum number (BPN). The size of the rubber sheet was 6.35 mm × 25.4 mm × 76.2 mm, and the test temperature was 20 °C. Equation (4) shows that the measured pendulum value BPN_t_ needs to be converted to the BPN_20_ at 20 °C when the pavement temperature is *t* °C.

(4) $$BPN_{20}=BPN_{t}+\Delta BPN$$

where *BPN*_20_ is the pendulum value when converted to 20 °C; *BPN*_*t*_ is the pendulum value measured at pavement temperature t; and Δ*BPN* is the temperature correction value, as shown in Table 4.

### 4.5. Flatness Test

The LXBP-6 pavement flatness meter (Hebei Jiehang Testing Instrument Co., Ltd., Cangzhou, China) was used to conduct the flatness test on the asphalt overlay. Before the operation, the wheel track strip on one side of the roadway was used as the standard position for the test; after the operation, the middle position of the rut on one side was taken as the test position.

### 4.6. Compaction Test 

The specimens were obtained by the core drilling method using a pavement coring machine (Cangzhou Kexing Instruments & Equipment Co., Ltd., Cangzhou, China), and the core samples were ≥100 mm in diameter. The test specimens were tested for compaction using an electronic balance and a constant temperature water bath (Shanghai Qianjun Scientific Instruments Co., Ltd., Shanghai, China). The compaction degree (*K*) is calculated according to Equation (5).

(5) $$K=\frac{\rho_{s}}{\rho_{0}}\times 100$$

where *K* is the compaction degree at the top of a certain pavement layer, %; *ρ*_*s*_ is the actual density of the asphalt mixture core sample, g/cm^3^; and *ρ*_0_ is the standard density of the asphalt mixture, g/cm^3^.

### 4.7. Permeability Test

The permeability test was carried out on the rutting slab using a pavement seepage meter (Shanghai Meiyu Instrument Technology Co., Ltd., Shanghai, China). Turn on the instrument switch; when the water drops to the 100 mL scale, the stopwatch to time was used immediately, and the scale of the instrument tube every 60 s was recorded until the water drops to the 500 mL scale. The pavement permeability coefficient (*C*_w_) is calculated according to Equation (6).

(6) $$C_{w}=\frac{V_{2}-V_{1}}{t_{1}-t_{2}}\times 100$$

where *C*_w_ is the pavement permeability coefficient, mL/min; *V*_1_ is the water volume at the first timing, mL; *V*_1_ is the water volume at the second timing, mL; *t*_1_ is the time of the first timing, s; and *t*_2_ is the time of the second timing, s.

### 4.8. Rutting Depth Test 

The LHHD-II pavement cross-sectional scale (Tianjin Ke’an Instrument Technology Co., Ltd., Tianjin, China) was used to test the rutting depth of test roads. For the same section, the maximum value of the result was taken as the rutting depth of the section.

## 5. Results and Discussion

### 5.1. Laboratory Performance of Asphalt Mixtures 

Before HWU and SBS-modified asphalt mixtures were applied to the test roads, their high-temperature stability, low-temperature crack resistance, moisture damage resistance, and skid resistance needed to be tested to ensure their pavement performance met the specification requirements. Three parallel specimens were prepared and tested in each test. The test results were also compared with the ultra-thin overlay porous asphalt concrete (PAC) prepared by Liu et al. [20]. The asphalt mixtures were classified into PAC-1 and PAC-2 according to the different proportions of coarse and fine aggregates. Since PAC-2 mixtures have a high proportion of fine aggregates and poor performance, only PAC-1 mixtures were compared in this study, and its gradation is shown in Table 5. Additionally, two different high-viscosity modifiers, type I and II, were used for the PAC mixtures. Hence, the mixtures used for comparison were referred to as PAC-1-I and PAC-1-II, respectively [20].

#### 5.1.1. High-Temperature Stability

**Figure 7:**
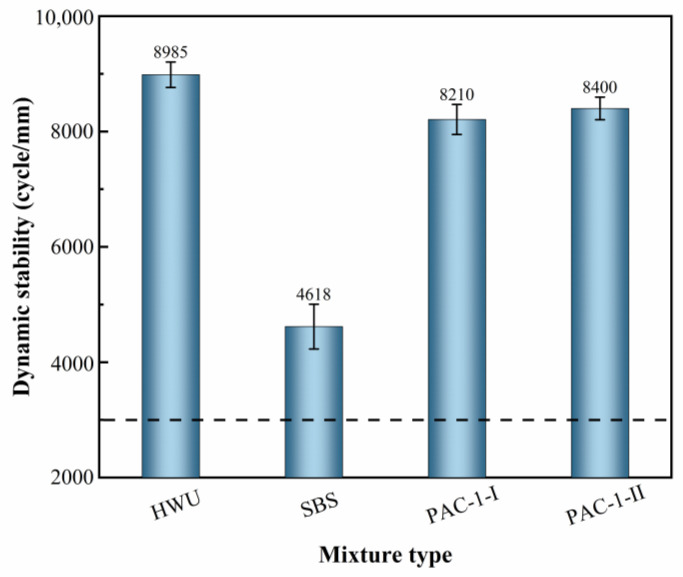
Rutting test results of the mixtures.

Figure 7 illustrates the results of the rutting test on asphalt mixtures. The DS of HWU and SBS, PAC-1-I, and PAC-1-II mixtures was 8985, 4618, 8210, and 8400 times/mm, respectively. It can be seen that the DS of the HWU asphalt mixtures was slightly higher than those of the PAC-1 mixtures and much higher than the SBS-modified asphalt mixtures. The results showed that the HWU asphalt mixture had the best high-temperature rutting resistance. The DS of SBS-modified asphalt mixtures was much lower than those of HWU and PAC-1 mixtures but still met the requirement of DS ≥ 3000 times/mm in the specification (JTG F40-2004) [23]. The specification (JTG F40-2004) stipulates that when the proportion of coarse aggregate (>2.36 mm) is higher than 45%, the asphalt mixture belongs to the coarse gradation. As shown in Figure 4, the coarse aggregate ratio in the HWU-10 gradation was 74.4%, indicating that both the HWU and SBS-modified asphalt mixtures were coarse gradations, which was consistent with the PAC-1 mixture (coarse aggregate ratio of 89.0%). Compared with fine-graded asphalt mixtures, coarse-graded asphalt mixtures had higher structural strength, which made them have better resistance to deformation under high-temperature conditions [20,21]. The fine aggregate ratio in the HWU asphalt mixture was higher than that of the PAC-1 mixture, which allowed more fine aggregate to fill in the voids formed by the coarse aggregates. The increase of fine aggregates played a vital role in improving the compactness and stability of the mixture. It was the main reason why the high-temperature rutting resistance of the HWU asphalt mixture was better than that of the PAC-1 mixture. As a whole, the HWU asphalt mixture had excellent high-temperature stability and could meet the requirements of engineering applications.

#### 5.1.2. Low-Temperature Crack Resistance

**Figure 8:**
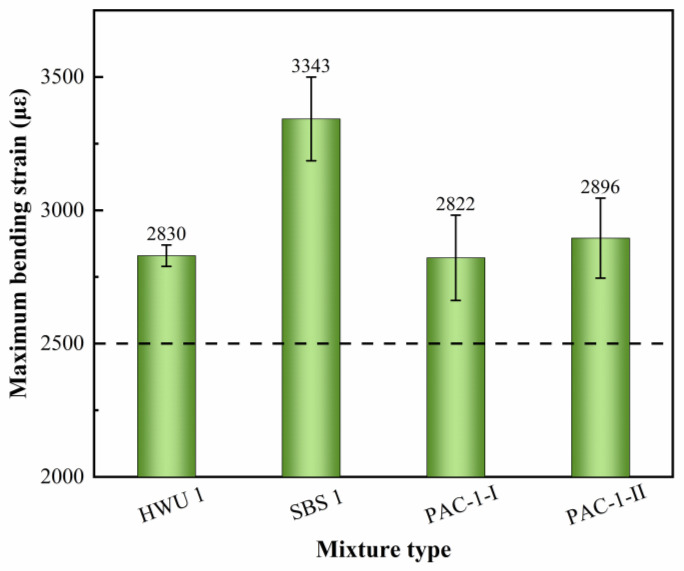
Bending beam test results of the mixtures.

The results of the bending beam test are shown in Figure 8. It can be seen that the SBS-modified asphalt mixture had the highest maximum bending tensile strain (*ε*_B_) of 3343 μ*ε*. The *ε*_B_ for the HWU asphalt mixture was 2830 μ*ε*, which was equivalent to the PAC-1-I and PAC-1-II mixtures. The *ε*_B_ of the HWU asphalt mixture was 15.3% lower than that of the SBS-modified asphalt mixture. It should be noted that despite the differences in *ε*_B_ of various asphalt mixtures, they were all able to meet the requirement of not less than 2500 μ*ε* in the specification (JTG F40-2004) [23]. The results showed that the HWU asphalt mixture had satisfactory low-temperature crack resistance but inferior to SBS-modified asphalt mixture. Furthermore, the low-temperature performance of the HWU and PAC mixtures was similar despite the use of different gradations, indicating that the asphalt binder was the main factor affecting the low-temperature cracking resistance of the mixtures rather than the gradation of the aggregates.

#### 5.1.3. Moisture Damage Resistance

**Figure 9:**
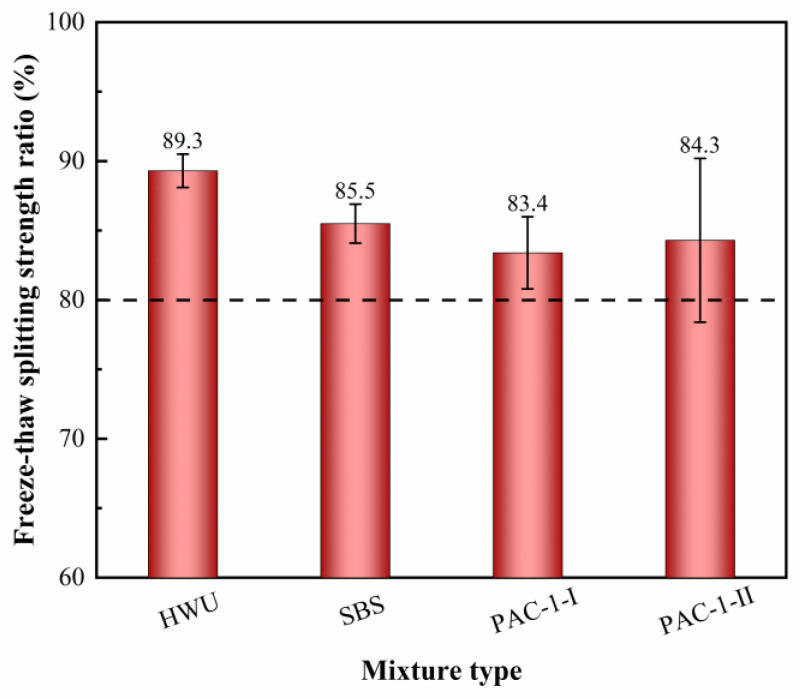
Freeze–thaw splitting tensile strength ratio results of the mixtures.

The results of the freeze–thaw splitting test are shown in Figure 9. It can be seen that the splitting strength of the mixtures after freeze–thaw cycling all showed different degrees of decrease. The reason was that the moisture entered the voids inside the structure of the mixture, which not only weakened the internal frictional resistance and embedded force between the aggregates but also reduced the adhesion between the asphalt binder and the aggregates [29]. The TSR for HWU, SBS, PAC-1-I, and PAC-1-II asphalt mixtures was 89.3%, 85.5%, 83.4%, and 84.3%, respectively. The results showed that the HWU asphalt mixture had the highest TSR, followed by the SBS-modified asphalt mixture. Affected by the low content of fine aggregates, the structure of the PAC-1 mixture had a sizeable internal porosity and was more susceptible to water damage, resulting in its lowest TSR. According to the requirements in the specification (JTG F40-2004) [23], the TSR of the asphalt mixture applied to road engineering should be not less than 80%. From Figure 9, it can be seen that the above four mixtures were able to meet the requirements, indicating that they had good moisture damage resistance. Aggregates with small particle sizes were more easily wrapped by asphalt to form a monolith, which helped to enhance the water damage resistance of the mixture [20]. Moreover, as mentioned in the rutting tests, the HWU and SBS-modified asphalt mixtures had a suitable fine aggregate content in the gradation used. This had a positive impact on improving the compactness and stability of the mixture, reducing the damage to the internal structure of the asphalt mixture by temperature and moisture, and making the mixture exhibit better moisture damage resistance.

#### 5.1.4. Skid Resistance

**Figure 10:**
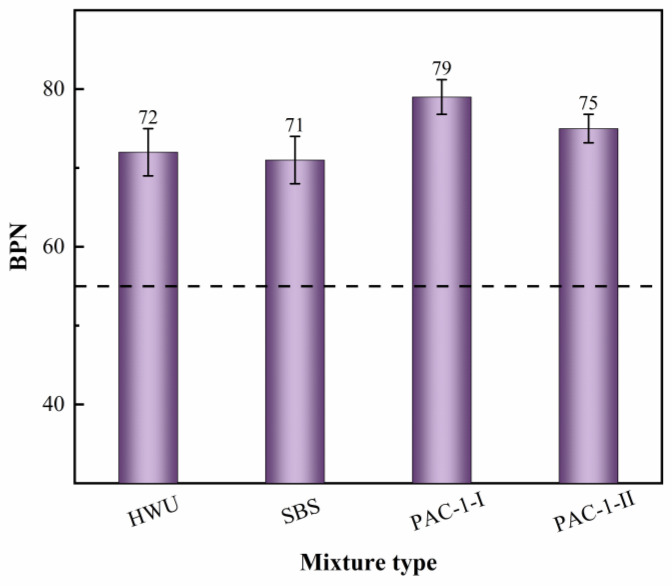
Pendulum test results of the mixtures.

Figure 10 provides the pendulum test results of the mixtures. The BPN for HWU and SBS-modified asphalt mixtures was 72 and 71, respectively. The BPN order of the mixture was: PAC-1-I > PAC-1-II > HWU > SBS. The “Technical Specifications for Maintenance of Highway Asphalt Pavement” (JTG 5142-2019) requires that the BPN of ultra-thin overlay asphalt mixtures should be not less than 55 [30]. The results showed that all of the above mixtures could meet the specification requirements and have good skid resistance. Notably, the PAC-1 mixture exhibited the best skid resistance, and the HWU asphalt mixture was comparable to the SBS-modified asphalt mixture. The reason was that the skid resistance of asphalt mixtures had a significant correlation with the aggregate gradation. In general, the higher the proportion of coarse aggregate, the rougher the macrotexture and the better the skid resistance of the asphalt mixture [31]. It can be seen from Figure 4 and Table 5 that, for the proportion of coarse aggregate in the mixture, the PAC-1 mixture was the highest, and the HWU asphalt mixture was the same as the SBS, which corresponded to their skid resistance.

### 5.2. Tests and Comparisons of Test Roads before and after the Operation

#### 5.2.1. Pavement Flatness

As shown from Table 6, the average values of the flatness of test roads 1, 2, and 3 before the operation were 0.58, 0.65, and 0.61 mm, respectively. The test road was susceptible to various extents of rutting on the pavement during operation due to vehicle loads and channelized traffic. As a result, all test roads showed an increase in pavement level after one year of operation, rising by 0.16, 0.07, and 0.11 mm to 0.74, 0.72, and 0.72 mm in that order. It is worth noting that, compared to the test roads 2 and 3 paved with HWU asphalt mixture, test road 1 paved with SBS-modified asphalt mixture showed the largest change in flatness before and after the operation. As demonstrated by the laboratory rutting test results, the DS of the SBS-modified asphalt mixture was 4618 times/mm, much lower than the 8995 times/mm of the HWU asphalt mixture. The poor high-temperature rutting resistance was the main cause of the highest variation in the flatness of test road 1. Furthermore, compared to test road 2, test road 3 showed a larger change in pavement flatness after one year of operation. The reason is that, as shown in Figure 3, the traffic volume of test road 3 was 23,300 pcu/d, reaching more than twice that of test road 2. Test road 3 was subjected to heavier vehicle loads, which aggravated the variation of pavement flatness. The “Inspection and Evaluation Quality Standards for Highway Engineering” (JTG F80/1-2017) requires the flatness of the pavement to be ≤1 mm [32]. It can be seen that the flatness of the three test roads before operation and after one year of operation satisfy the regulations. The results showed that the HWU asphalt mixture prepared with high-strength-modified asphalt binder had excellent high-temperature deformation resistance, and the ultra-thin overlay paved with it on the test road had satisfactory rutting resistance and flatness.

#### 5.2.2. Skid Resistance

**Figure 11:**
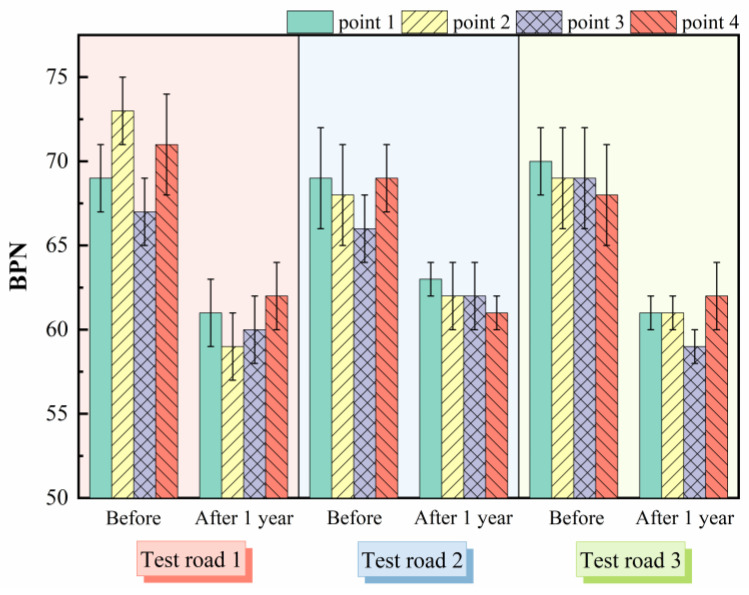
Pendulum test results before and after test roads operation.

The pendulum test results before and after the operation of the test roads are shown in Figure 11. The BPN before the operation for the three test roads was 70, 68, and 69, which were similar to the results of the indoor tests. It can be seen that after a period of operation, the BPN of the test roads all showed a significant decrease, indicating that their skid resistance had been reduced. With the operation of the test roads, the exposure of the vehicle tires to the aggregates acts as a polishing and abrasion effect on the aggregates of the pavement [33]. As a result, the abundant texture of the pavement surface was gradually worn away during operation, resulting in a gradual decline in skid resistance. It is noteworthy that the BPN reduction was higher for test roads 1 and 3 than for test road 2. The reason is that the traffic volumes of test roads 1 and 3 were 17,108 and 23,300 pcu/d, which were 1.54 and 2.09 times of test road 2. More vehicle tire–road friction exacerbated the attrition of aggregates. The skid resistance of the three test roads after one year of operation could still meet the requirement of the standard (JTG F80/1-2017) that the BPN of pavement is not less than 55 [31]. It is important to note that a decline in skid resistance will have a negative impact on driving safety. Thus, it is particularly critical to test the skid resistance of the pavement during operation and to maintain it on time to ensure the safety of the roadway.

#### 5.2.3. Compaction Degree

**Figure 12:**
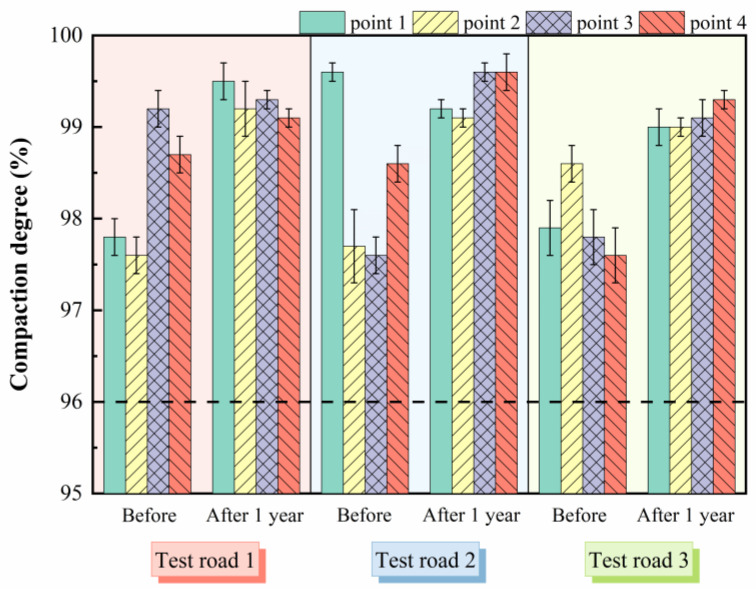
Compaction degree test results before and after operation of the test roads.

A high compaction degree plays an essential role in reducing the occurrence of asphalt pavement defects and ensuring traffic safety. Figure 12 illustrates the compaction test results of different test roads before and after the operation. The specification (JTG F40-2004) requires that the compaction of the pavement should be not less than 96% during construction. It can be seen that the average compaction degrees of the three test roads before the operation were 98.2%, 98.4%, and 98.0%, respectively, all of which can meet the specification requirements. Meanwhile, it also showed that the asphalt mixture with HWU-10 gradation was easy to be compacted during construction, which enhanced the workability of the mixture. After one year of operation, the pavement compaction degrees of the test roads were all over 99%. This was due to the long-term vehicle loading effect on the asphalt mixture to play a re-compaction effect so that the pavement structure had better compactness. The results showed that the ultra-thin asphalt overlays after operation have higher compaction, which effectively improved the compactness and structural stability of the pavement and played a positive role in reducing the occurrence of pavement defects [34].

#### 5.2.4. Permeability Performance

**Figure 13:**
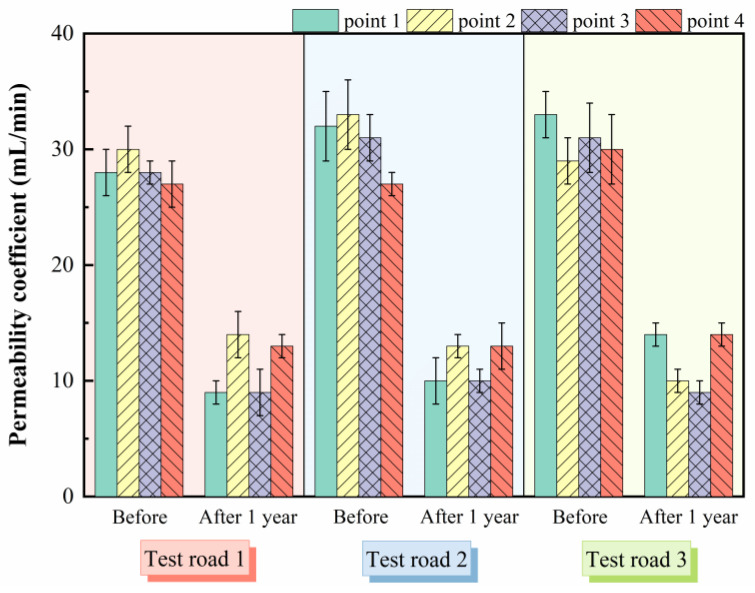
Permeability test results before and after operation of the test roads.

The poor anti-permeability of asphalt pavements will result in water penetrating asphalt mixtures. In the role of wheel load generated by the repeated cycle of water pressure and negative vacuum suction, moisture gradually penetrates deep into the interface of asphalt binder and aggregate, reducing the adhesion of asphalt and loss of cohesion [35,36]. In this case, the asphalt mixture is prone to spalling, loosening, and other defects, resulting in a decline in durability. Consequently, it is necessary to ensure that the *C*_w_ of the asphalt mixture meets the specification requirements. The standard (JTG F80/1-2017) requires that the *C*_w_ of asphalt pavement should be less than 80 mL/min [32]. As shown in Figure 13, the *C*_w_ of the test roads before the operation was lower than the requirements, indicating that the paved ultra-thin asphalt overlays had a good anti-permeability performance. After one year of operation, the *C*_w_ of the test roads was significantly reduced, with the highest value being only 14 mL/min, and the anti-permeability performance was further improved. As mentioned above, the action of the traffic load after operation increased the compaction degree of the pavement and made it more difficult for moisture to enter the interior of the mixture structure, ensuring the water damage resistance of the ultra-thin asphalt overlays. Meanwhile, the *C*_w_ of the three test roads was always similar both before and after one year of operation, which indicated that the influence of the bonding material on the water anti-permeability performance of the mixtures was not significant at the same aggregate gradation.

#### 5.2.5. Rutting Depth

**Figure 14:**
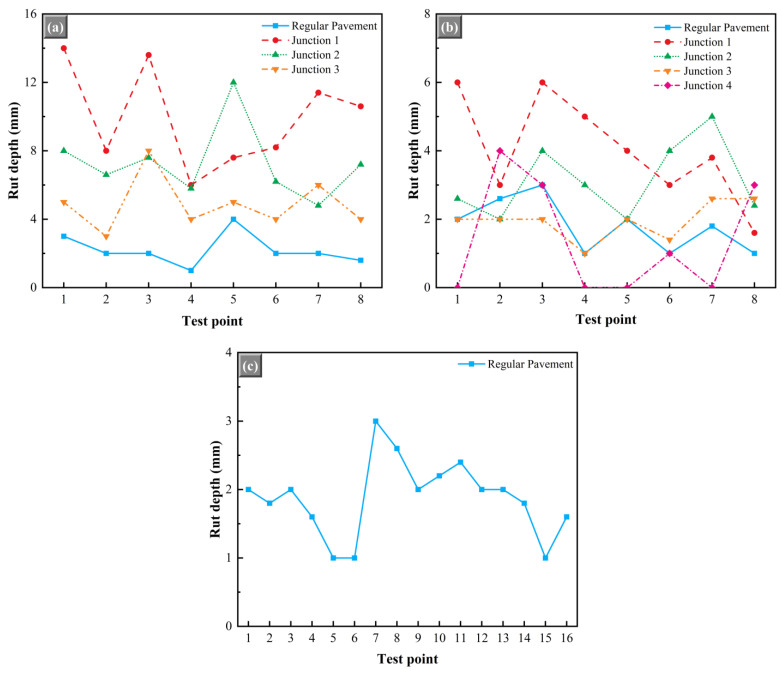
Test results of rutting depth: (**a**) test road 1; (**b**) test road 2; (**c**) test road 3.

The pavement other than the junctions was designated the regular pavement on the test road. In the rutting depth test of asphalt pavements, it should be noted that the vast majority of conventional pavements were considered to be almost rut-free. Hence, in contrast to the equal conventional equal inspection according to the length of the road, this study selected local inspection at the location of the large rutting in the pavement in a targeted manner. For the junctions, the test situation was similar to the above, and only the most prone to rutting in front of the road stop line was selected for testing. The test results are shown in Figure 14. 

**Figure 15:**
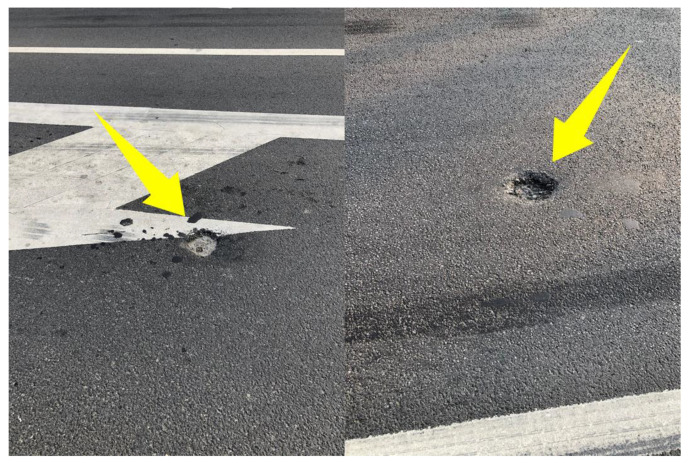
Pothole defects at the junctions of test road 1.

Figure 14a demonstrates the rutting depth test results for the ultra-thin overlay of test road 1. It can be seen that even if only the locations with large ruts were selected for local inspection, the rutting depth of the vast majority of measurement points on test road 1 was below 4 mm, and it was in good overall condition. In contrast, due to traffic control, traffic signals, and the inevitable presence of vehicle braking, the rutting defects at junctions were more severe than on regular pavements [37]. Particularly, for junctions 1 and 2, the average rutting depth reached 7.3 and 9.9 mm, and the rutting depth at the individual test points exceeded 12 mm. As shown in Figure 15, the monitoring points with large rutting depths were located at pothole defects, which were mainly caused by the local presence of water damage. For junction 3, the maximum and average values of rutting depth were 8 mm and 4.9 mm, respectively, which showed the best performance among the three junctions.

The results of the rutting depth test for test road 2 are shown in Figure 14b. The results showed that the rutting depth of regular pavement of test road 2 mainly was below 2 mm; even the rutting at the most prominent place was only 3 mm, which indicated that the ultra-thin overlay paved with HWU asphalt mixture showed good rutting resistance. The average rutting depths of junctions 1, 2, 3, and 4 were 4.1, 5.1, 3.9, and 2.0 mm, respectively, and the overall performance was significantly better than that of test road 1. Different from test road 2, the ultra-thin overlay of test road 1 was selected from the SBS-modified asphalt mixture, which showed inferior high-temperature stability and moisture damage resistance to the HWU asphalt mixture in laboratory performance tests, resulting in more rutting and water damage under the influence of vehicle loads and rainfall.

The rutting depth test results for test road 3 are shown in Figure 14c. It should be noted that this test road does not intersect with other roads, so only its regular pavement was measured. The test results showed that there was no monitoring point with abnormal rutting depth on test road 3. Its average rutting depth was 1.88 mm, and the most profound depth was only 2.6 mm. Under the condition of maximum traffic volume, its rutting depth was much lower than that of test road 1 after one year of operation, which showed excellent high-temperature stability.

The pavement flatness, skid resistance, compaction degree, anti-permeability performance, and rutting depth were compared and analyzed before and after the operation of the test roads. The results showed that the asphalt mixtures using HWU-10 gradation have high compaction degrees and excellent workability. Test road 1 paved with the SBS-modified asphalt mixture showed apparent rutting and pothole defects after one year of operation, which was related to the poor laboratory high-temperature rutting resistance and moisture damage resistance of the SBS-modified asphalt mixture. The test roads paved with the HWU asphalt mixture, especially test road 3, showed excellent high-temperature rutting and moisture damage resistance with no significant pavement defects under the highest traffic volume. The application of a high-strength-modified asphalt binder and the addition of polyester fibers to the mixture played a positive role in ensuring the excellent pavement performance of the HWU asphalt mixture. However, it was found that defects at junctions were significantly more severe compared to regular pavements, and further measures to enhance the serviceability of junction pavements are necessary for future research. Importantly, the excellent pavement performance of the HWU asphalt mixture indicated that it has good application and promotion value.

## 6. Conclusions

In this study, the HWU asphalt mixture was prepared using a high-strength-modified asphalt binder and an optimized HWU-10 gradation and then used for paving the test roads. A series of tests were conducted to test the laboratory performance of the HWU asphalt mixture as well as the field performance before and after the operation of the test roads and compared with the SBS-modified asphalt mixture. The main conclusions are summarized as follows:

1. The laboratory performance of the HWU asphalt mixture met the specifications. The use of a high-strength-modified asphalt binder and polyester fibers to the mixture contributed to the improved pavement performance of the HWU-modified asphalt mixture, which had better high-temperature stability and moisture damage resistance than that of the SBS-modified asphalt mixture.
2. The asphalt mixture prepared with HWU-10 gradation was easily compacted during the test road paving. It showed good construction workability, which played a positive role in improving the compactness of the pavement and reducing the occurrence of defects.
3. After one year of opening traffic, the flatness and skid resistance of the test roads decreased while the compaction degree, anti-permeability, and rutting depth increased due to the influence of vehicle load. The poor high-temperature rutting resistance and moisture damage resistance of the SBS-modified asphalt mixture resulted in rutting and potholes on the test road surface. Compared with the SBS-modified asphalt mixture, the ultra-thin overlay paved with HWU asphalt mixture showed excellent performance degradation resistance and rutting resistance, which has potential for promotion and application in pavement preventive maintenance.
4. This study only investigated the laboratory and field performance of asphalt mixtures and did not fully consider the effects of traffic volume and vehicle load on the test road pavement performance. In-depth analysis on asphalt binders and measures to ensure uniform dispersion of polyester fibers in the mixture and enhance the performance of road intersection pavements will be conducted in the future.

## Figures and Tables

**Table 1:** Physical properties of high-strength and SBS-modified asphalt binders.

Items		High-Strength Asphalt Binder		SBS-Modified Asphalt Binder		Test Standards
		Results	Specifications	Results	Specifications	
Penetration (25 °C, 100 g, 5 s) (0.1 mm)		24	20–40	57	40–70	T 0604-2011
Penetration index (PI)		0.1	≥−0.4	0.04	−0.2–1.0	
Ductility (5 °C, 5 cm/min), cm		—	—	35	≥25	T 0605-2011
Softening point (°C)		76.7	≥60	80	≥70	T 0606-2011
Kinematic viscosity (135 °C) (Pa.s)		2.8	≤3	2.6	≤3	T 0625-2011
Dynamic viscosity (60 °C) (Pa.s)		6400	≥4000	30,000	≥20,000	T 0620-2000
Flash point (°C)		320	≥230	315	≥245	T 0611-2011
Storage stability, 48 h softening point difference (°C)		1.2	≤2.5	1.5	≤2.5	T 0661-2011
Rotating film heating (163 °C, 5 h)	Mass variation (%)	−0.15	−1.0–1.0	−0.16	≤0.5	T 0609-2011
	Penetration ratio (%)	75.4	≥65	75	≥65	T 0604-2011
	Ductility (5 °C, 5 cm/min) (cm)	—	—	22	≥15	T 0605-2011

**Table 2:** Physical properties of polyester fiber.

Items	Results	Specifications	Test Standards
Tensile strength (MPa)	763	≥550	GB/T 14337-2008
Elongation at break (%)	32.4	30 ± 9	
Color	White	White	—
Melting point (°C)	255	≥230	FZ/T 01057.6-2007
Diameter (μm)	19.9	20 ± 4	—
Density (g/cm^3^)	1.38	1.36–1.40	GB/T 14335-2008

**Table 3:** Test standards for mixture and pavement performance tests.

Test	Specifications	Test Standards
Rutting test	JTG E20-2011	T0719-2011
Bending beam test		T0715-2011
Freeze–thaw splitting tensile strength test		T0729-2011
Pendulum test	JTG 3450-2019	T0964-2008
Flatness test		T0932-2008
Compaction test		T0924-2008
Permeability test		T0971-2019
Rutting depth test		T0973-2019

**Table 4:** Temperature correction values for the pendulum test.

Temperature (°C)	0	5	10	15	20	25	30	35	40
Temperature correction value Δ*BPN*	−6	−4	−3	−1	0	+2	+3	+5	+7

**Table 5:** Gradation of PAC-1 mixtures.

Mesh Size (mm)	9.5	4.75	2.36	1.18	0.6	0.3	0.15	0.075
Passing percentage (%)	100.0	17.7	11.0	7.0	6.8	6.1	5.1	4.0

**Table 6:** Flatness test results of the test roads before and after the operation.

Test Road Number	Test Road 1		Test Road 2		Test Road 3	
	Before	After 1 Year	Before	After 1 Year	Before	After 1 Year
Number of detection points	100	20	116	59	92	20
Mean value of flatness (mm)	0.58	0.74	0.65	0.72	0.61	0.72

## Data Availability

All data, models, and code generated or used during the study appear in the submitted article.

## List of Abbreviations

**Abbreviations**	**Phrases**
HWU	High-Workability Ultra-thin
HUFC	High-ductility Ultra-thin Friction Course
UTWC	Ultra-thin Wear Course
SMA	Stone Matrix Asphalt
SUP	Superpave
SBS	Styrene-butadiene-styrene
DS	Dynamic Stability
UTM	Universal Testing Machine
TSR	Tensile Strength Ratio
BPN	British Pendulum Number
HWU	Porous Asphalt Concrete
